# Impact of Pharmaceutical Impurities in Ecstasy Tablets: Gas Chromatography-Mass Spectrometry Study

**Published:** 2016

**Authors:** Amir Jalali, Amir Hatamie, Tahere Saferpour, Alireza Khajeamiri, Tahere Safa, Foad Buazar

**Affiliations:** a *Dept of Pharmacology and Toxicology, Faculty of Pharmacy and Toxicology Research Center, Ahvaz Jundishapur University of Medical Sciences, Ahvaz, Iran.*; b *Dept of Chemistry, Faculty of Sciences, Shahid Chamran Universitry of Ahvaz, Ahvaz, Iran.*; c *Dept of Chemistry, Azad University of Medical Sciences, Rasht, Iran. *; d *Dept of Pharmacology and Toxicology, Faculty of Pharmacy, Shahid Beheshti University of Medical Sciences, Tehran, Iran. *; e *Dept of Chemistry, Khoramshahr University of Marine Science and Technology, Khoramshahr, Iran.*

**Keywords:** Gas chromatography–Mass Spectrometry (GC–MS), Identification, Determination, MDMA, Iran

## Abstract

In this study, a simple and reliable method by gas chromatograph–mass spectrometry (GC–MS) was developed for the fast and regular identification of 3, 4-MDMA impurities in ecstasy tablets. In so doing, 8 samples of impurities were extracted by diethyl ether under alkaline condition and then analyzed by GC–MS. The results revealed high MDMA levels ranging from 37.6% to 57.7%. The GC-MS method showed that unambiguous identification can be achieved for MDMA from 3, 4-methylenedioxyamphetamine (MDA), Amphetamine (AM), methamphetamine (MA) and ketamine (Keta) compounds, respectively. The experimental results indicated the acceptable time window without interfering peaks. It is found that GC-MS was provided a suitable and rapid identification approach for MDMA (Ecstacy) tablets, particularly in the Forensic labs. Consequently, the intense MDMA levels would support the police to develop a simple quantification of impurity in Ecstasy tablets.

## Introduction

 A worldwide consumption of the well-known synthetic drugs (club drugs) increased annually. In the most countries, there has been considerable growing in recent years in use of “Club Drugs mainly as tablets or capsules in the young and youngster generation (1-4). It is available in various forms on the drug market, mainly as tablets, capsules or powder, all called ecstasy. 

 The Ecstasy tablets mostly contain amphetamine-type stimulants including 3, 4-methylenedioxymethamphetamine (MDMA), 3, 4-methylenedioxyamphetamine (MDA), methamphetamine (MA) and ketamine (Keta), etc (4,5). The term is used more generally for all amphetamine type substances sold in form of tablets in the illicit market. Amphetamines and other related derivatives are powerful stimulants of the central nervous system and one of the most highly addictive drugs. 

 The majority of people take MDMA orally, most often in tablet or capsule form. MDMA poisoning features are due to release of endogenous catecholamines (particularly norepinephrine and dopamine) and block their reuptake into presynaptic vesicles. MDMA produce profoundly positive feelings and empathy for others, that it eliminates anxiety. Furthermore, MDMA overdose is characterized by persistent psychosis, high blood pressure, panic attack, and in more severe cases, loss of consciousness, seizures and a marked rise in body temperature. In some cases, at high doses, it can cause convulsions, loss of consciousness, coma, and hyperthermia respectively (6-8).

 Due to the widespread abuse of amphetamine, drug testing for amphetamines is routinely done in forensic toxicology. So, introduction of new and fast determination method is essential (9). Impurities in illicit methamphetamine tablets have been investigated and/or profiled by researchers around the world. Detailed impurity information has been reported on the drugs seized in countries such as Australia (10), Thailand (11), Philippines (12), Korea and Japan respectively (13). 

 Hence, a rapid, precise and reproducible methodology is required for the quantitative and qualitative determination of drugs for forensic science. Several methods such as gas chromatography (GC) (2), GC-mass spectrometry (GC-MS) (3, 5-6), high-performance liquid chromatography (HPLC) (14, 15), capillary electrophoresis (CE) (1) and IR Spectroscopy (16) have been used. These methods are time-consuming, especially concerning diagnosis, since often require the use of more ones analytic method to find the amphetamine drugs. GC-MS method is safer because it doesn’t require exposure of the person to the amphetamine, as it is done in vitro with available sample. Another advantage is that the technique is trouble-free to do and may be easily available in health centers, as opposed to other methods that are more expensive (such as LC–MS–MS (17)) and therefore not accessible to all diagnostic centers including local antinarcotics police. A general disadvantage of quantitation based on in vivo detection is relatively lower abundance for ones of the chemical derivatization of samples resulting lower coverage. 

GC-MS methods are often preferred for quantitative determination. To the best of our knowledge, the use of different methods did not provides sensitive and selective detection of amphetamine containing tablets, with the exception of 3,4-MDMA. 

 This study was aimed to describe a GC–MS method for impurity profiling, after a simple liquid–liquid extraction. The first aim was to develop a fast, routine, quantitative and qualitative GC-MS method for confirmation of the presence of 3, 4- MDMA in the sized drugs. The ecstasy tablets seized in Ahwaz-Iran were examined and the data matrices were analyzed. This method evidently makes possible a simple identification of impurity in Ecstasy tablets. The second aim was to study GC-MS pattern of 3.4-MDMA to detect it among the compounds. 

## Experimental

*Samples*

 All ecstasy samples discussed were obtained in tablet forms. The samples were confiscated by the Ahwaz-Iran antinarcotics police. The 8 random samples were categorized into 4 groups and analyzed. The tablets weighed from 0.20 g to 0.39 g (*ex.* 0.3 g= 300mg) with diameter from 6 mm to 8 mm. 

*Chemicals and reagents*

 Authentic standards of 3, 4-methylenedioxymethamphetamine (MDMA), 3, 4-methylenedioxyamphetamine (MDA), Amphetamine (AM), methamphetamine (MA) and ketamine (Keta) were purchased from Sigma Chemical Co. MDMA-d5 as an internal standard was purchased from Sigma–Aldrich. Diethyl ether (anhydrous, >99%) and sodium carbonate was purchased from Merck (Darmstadt, Germany), and was used as the extraction solvent. All standard solutions were prepared with doubly distilled water. They were stored in the refrigerator at 4 ºC. 

*GC–MS instrument and conditions*

 The analyses were performed on an Agilent GC system. The GC was equipped with a HP5-MS capillary column (25 m, i.d :0.20 mm., film thickness 0.25 µm) and with using an Agilent 7683 automatic liquid sampler, 1 µL sample was injected under split less mode. Helium was used as the carrier gas at a flow rate of 0.9 mL/min. 

 The GC–MS system was operated under following conditions: The following oven temperature program was used to run all the samples: initial temperature 90 ºC held for 1 min; 10 ºC/min to 280 ºC; interface temperature was 280 ºC, while the temperatures of MS quadruple and ion source were 150 ºC and 230 ºC, respectively. Then the final temperature held for 15 min. The MS system was operated in electron impact mode. EI was used as ionization mode at 70 eV. The measurements were carried out in the selected ion monitoring mode in three time windows. MS scanning was performed at the range of 35–450 amu.

*Sample preparation and* *Qualitative analysis*

 GC-MS was used for determination of chemical profile of the tablets. A total of 8 samples of ecstasy tablets were studied from each kind of four provided samples, collected randomly from seizures at 2010 year. These samples were chosen from more than 120 samples of ecstasy tablets seized in local antinarcotics police. These samples were divided into four groups according to their physical and appearance characteristics (such as shape, break line, color, weight, diameter and thickness). Ecstasy tablets were ground and homogenized. Then, 5 mg of the powdered tablets was dissolved in 2 mL distilled water. 150 μL of this solution was mixed with 100 μL of d5-MDMA solution (1 mg/mL water) as an internal standard. To this solution was added 750 μL of 10% sodium carbonate solution (final pH about 10.0). The mixing was followed by addition of 2 mL pre-distilled diethyl ether. The solution was mixed 10 min and centrifuged at 3500 rpm. The ether layer was then separated and was transferred into a glass insert of GC micro vial for automatic sampling and dried. The residue was dissolved in 500 μL methanol and 1 μL of this solution was injected into GC by auto sampler. GC–MS analysis was carried out to identify the impurities. In order to avoid impurity degradation, the extracts were prepared and injected on the same day.

## Results and discussion 

 Due to the increasing number of drug cases, as well as the widening globalization of illicit drugs, law enforcement agencies worldwide have adopted the strategy of profiling of drug impurities. They consist of a large variety of active ingredients and some contain a mixture of MDMA and one or more amphetamine-type drug(s) such as ketamine with apoptotic and neurodegenerative activities (18). Amphetamine drugs are strongly controlled in most countries. Analytical information derived from the analysis of the illicit drugs, is important for legal and intelligence purposes. Liquid–liquid extraction (LLE) is a classical technique that has been often used for carrying out the extraction of many compounds from various kinds of samples. Most laboratories prefer using liquid–liquid extraction for sample preparation. GC–MS is recently the most used method for the determination of drugs which allows working with complex matrixes leading to high specificity along with sensitivity. 

*Identification of impurities in illicit tablets seized*

**Figure 1:**
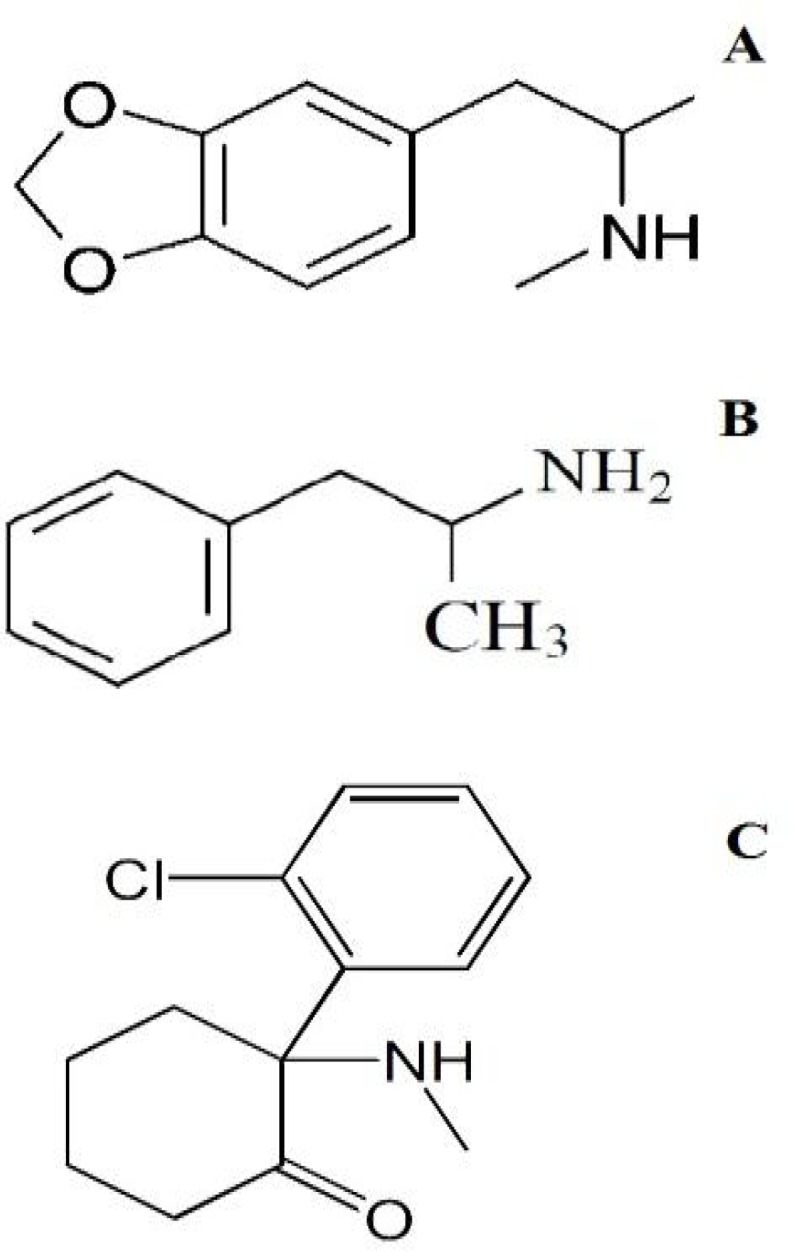
Structure of MDMA (A) Metamphetamine (B) and Ketamine (C).

**Figure 2:**
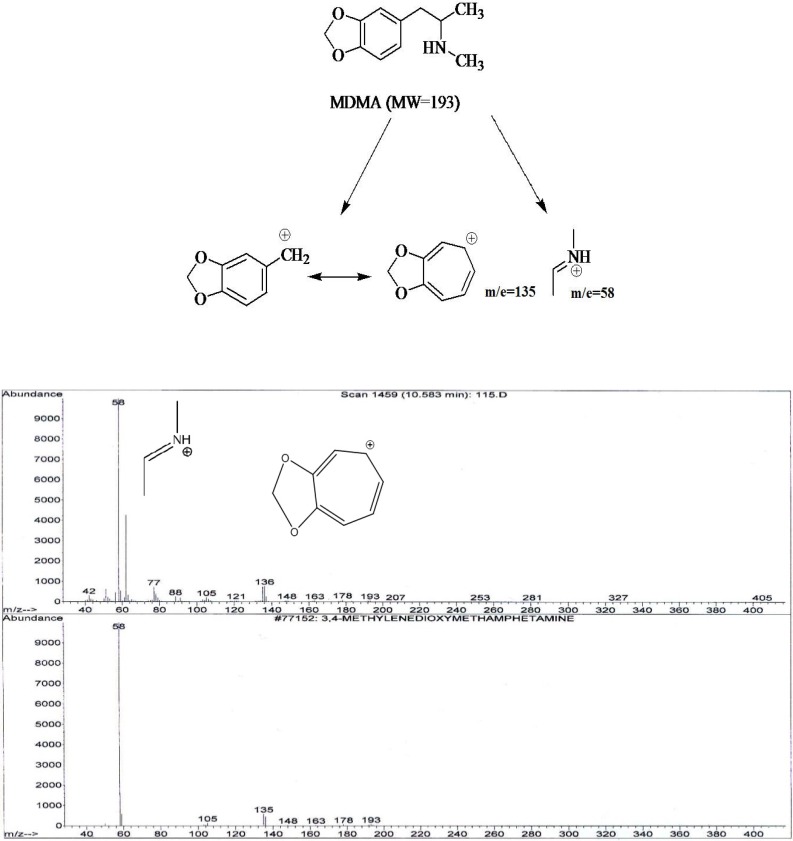
The proposed fragmentation for monitoring of 3, 4- MDMA and typical MS spectra profile for the analysis of tablet sample A and internal standard (down).

**Figure 3:**
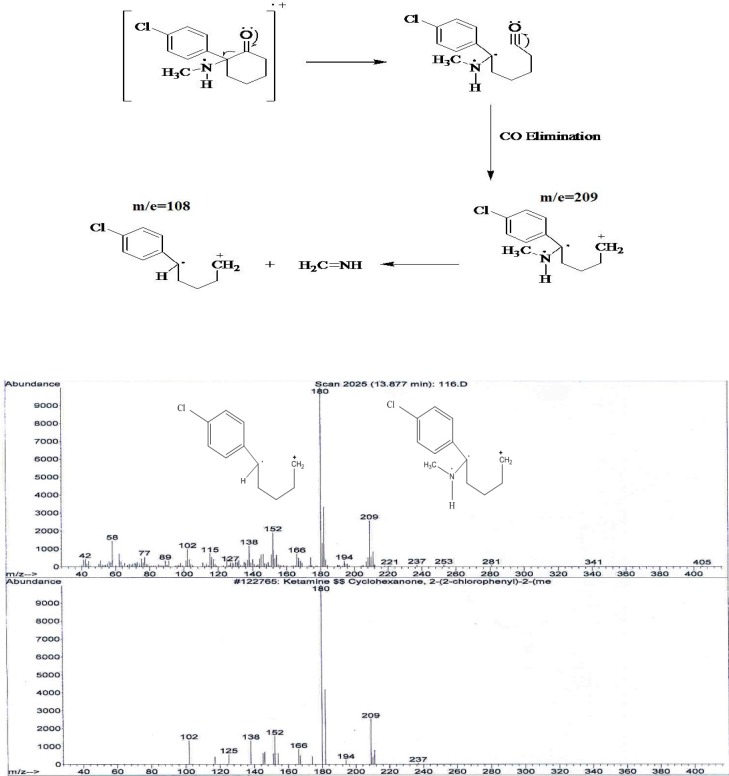
The proposed fragmentation for monitoring of Ketamine and typical MS spectra profile for the analysis of tablet sample B (up) and internal standard (down).

 GC-MS method showed full advantage of the high resolution for impurities using multiple points of selectivity for identification based on the match of retention time and mass spectrum of an unknown peak with that of the standard (Figure 1, 2 and 3). Quantification was performed by construction of 5-point calibration curves (2-40 ng/mL) that prepared by following concentrations: 2, 5, 10, 20 and 40 ng/mL, respectively. The internal standard method was used to construct the calibration curve. Acceptable linear regression was obtained for calibration curves (Table 1).

 In order to show the specificity of GC-MS method in Amphetamines measurement, the determinations were carried out in two series; with and without solvent. Only a peak was seen in solution. This peak was not observed in solvent. This feature shows the selectivity of GC-MS method in the amphetamine determination. 

**Figure 4:**
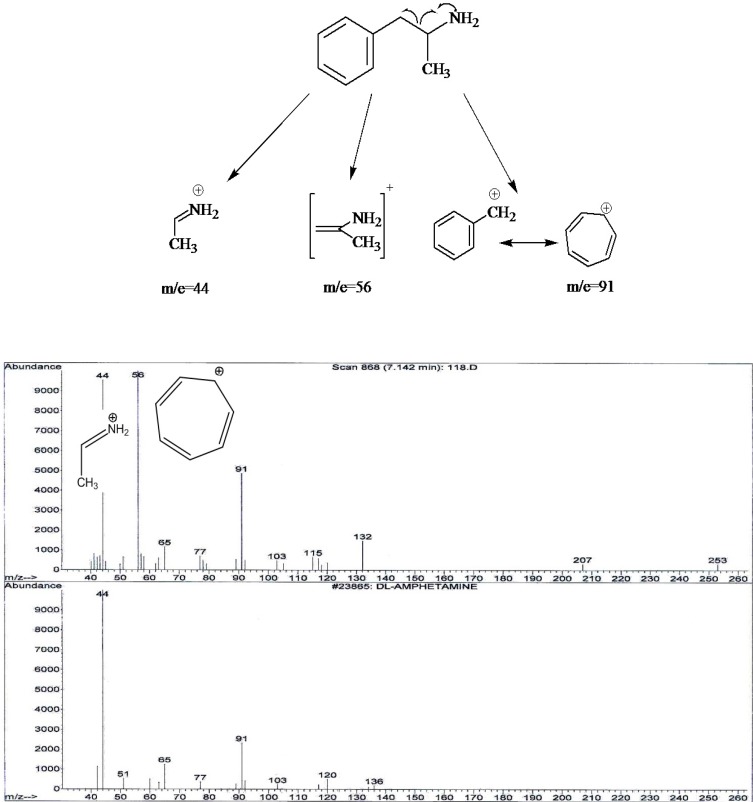
The proposed fragmentation for monitoring of Metamphetamine and typical MS spectra profile for the analysis of tablet sample C (up) and internal standard (down).

 After construction calibration curves for target compounds, the proposed procedure described above was applied to determine the target analytes in tablet samples. Typical MS spectrum of the diethyl ether extracts of drug samples are shown in Figure 2-4. With using information are listed in Table 1, the amount of present compounds were calculated. Table 2 is shown data of amount impurities found, as a result of GC–MS analysis. The amount of MDMA was different in all samples. It is noteworthy that one tablet (D) contained no MDMA and ketamine. 

It was showed that fragmentation of 3, 4- MDMA mainly by a-cleavage, yielding propylimine as a base peak (m/z=58) and methylenedioxyphenyl cation and radical cation (m/z = 135,136, respectively) (17). The mass spectra of the studied compounds showed sharp and strong peaks at m/e= 58, m/z=135, m/z= 180, m/e=209, m/z=44 and m/z=91. The reproducibility of the retention times was also determined using a concentration range (2-40 ng/mL). 

 The sensitivity of the MDMA analysis and the specificity of peaks are improved (3, 5-6). The peaks of derivatives are particularly desirable when the mass spectrum of the underivatized molecules is of low diagnostic value. Most underivatized molecules have fragmentations of low m/z ratio and low intensity. Derivatizations of MDMA usually produce fragmentations of higher m/z ratio and value, since they are not affected by interfering background such as column bleed or other contamination. 

 Multi-drug combinations were found only in one tablet sample. Among the 8 tablet sampled, 6 contained MDMA and 2 tablets (C) contained ketamine in addition to MDMA. 

 In this study, the range of tablets weight was 0.20 to 0.39 g (mean 0.3g=300mg) with diameter from 6 mm to 8 mm. The range of MDMA content was 0-58 percent (mean 90 mg). The tablet weight usually ranges from 40 to 140 mg. The content is differing regionally. The average MDMA in Europe was about 60-70 mg in the mid-1990s. 

The main results are followings: 

1) In the spectrum of tablet A two peaks m/e=58 and m/e=135 were seen. MDMA Identification was accomplished by comparing the retention time and mass spectrum of tablet A with standard spectrum. 

2) In the spectrum of tablet B also two main peaks with 10.6 minute and 13.8 minutes retention times were observed. Comparing the retention time 10.6 minute of mass spectrum of tablet B with standard spectrum show MDMA presence. The existence of two peaks m/z=180 and m/e=209 show keta presence. 

3) In tablet C chromatogram as well a peak in 10.6 minute retention time was seen. MDMA identification was accomplished by comparing the retention time and mass spectrum of standard spectrum.

4) In chromatogram D, no peak was seen in 10.6 minute retention time. Two peaks in m/e=44 and m/e=91 were seen. Amphetamine identification was accomplished by comparing the retention time at 1.7 minute and peaks with standard spectrum. 

 Profiling of seized ecstasy tablet in Iran was determined previously by liquid Chromatography-Mass Spectrometry (LC-MS) technique. The MDMA content in tablet samples were ranging from 60–180 mg. The range of tablets was 96-308 mg. The MDMA levels were ranging from 58.4% to 62.4% (19). The results of this study showed MDMA levels ranging from 37.6% to 57.7%. This study also demonstrated that the major metabolite was MDMA and no relative correlation between the tablets weight and MDMA contents. 

 GC technique is a fast analysis that recently coupled with MS. The GC method is not suitable for samples requiring extensive derivitization, as well as for complex samples such as seized samples for an effective separation of the large number of ingredients. The throughput and coverage of unknown samples were significantly improved by this coupling. The efficiency of the extraction process and the identity of MDMA peak were verified (20). LC-MS/MS is a widely used analytical tool for a broad drug screening (21). This method requires long run times . These times are less in GC-MS. GC-MS provide an appropriate method for large numbers of chemical including abuse drugs in a complex mixture. This technique unifies the separation power and sensitivity of a GC-FID with the analyte specificity of a spectroscopic technique. Therefore this method is able to provide highly specific spectral data on individual compounds in a complex mixture of compounds without prior separation. 

 The main achieving goals of the present study were minimizing cost, maximizing throughput, and/or efficiency. The present study showed the simultaneous quantification of amphetamine, 3,4-methylenedioxymethamphetamine (MDMA), Amphetamine (AM) and ketamine in tablets. The results indicated that for the target analytes studied, the GC-MS analysis was as precise, accurate, and specific as the LC-MS method.

 In this research, we used a simple liquid–liquid extraction prior GC-MS analysis. The sample preparation procedures prior to GC include several steps. In order to obtain high extraction recoveries of the drugs, some variables such as pH, extraction time and organic solvent were investigated and optimized. The method was optimized following a one-at-a time variable approach using the peaks area as analytical signals. 

 This study has demonstrated the successful application of GC-MS method for the quantitative and qualitative determination of the multiple illicit drugs. This method is a rapid detective tool in the clinical emergency management. All experiments were applicable in less than half-hour. The experiments require low levels of the sample. The preparation method is minimal and simple. 

**Table 1:** Standard curves parameters prepared through concentrations: (2-40 ng/mL)

Analyte	Mass ions	Linear equation	R
MDMA	58.135	y = 0.109x -0.068	0.9998
AM	91.44	y = 0.118x -0.070	0.9991
Keta	180.209	y = 0.113x -0.0900.9990	0.9990

**Table 2:** Impurities detected in Ecstasy tablet samples

Tblet Sample	MDMA(%)	AM(%)	Ket(%)	MDEA	Metampamine
A	48.0	N/A	N/A	N/A	N/A
B	57.7	N/A	Lower than 1.0	N/A	N/A
C	37.6	N/A	N/A	N/A	N/A
D	N/A	Lower than 1.0	N/A	N/A	N/A

N/A : not observed

## Conclusions

 This work not only provides information about MDMA content of ecstasy tablets, but also develops a new method for the fast and regular identification of 3, 4-MDMA impurities in ecstasy tablets. 

 Chemical profiling is able to provide more reliable information than the physical characteristics of the tablets. Also, impurity profiling could reveal the hidden information on the clandestine MDMA laboratory. The Information about the impurities in methamphetamine allowed identification of the drug synthetic routes. We are actively pursuing the identities of the unknown impurities via synthetic approach and will report the outcomes of our efforts in next publication.
